# Effect of magnesium reduction on the oxygen content of pickling niobium powder

**DOI:** 10.1038/s41598-021-94578-7

**Published:** 2021-07-22

**Authors:** Jingfeng Wang, Yue Zhang, Fang Liu, Qingkui Li

**Affiliations:** 1 Zhengzhou Railway Vocational and Technical College, Zhengzhou, 450000 Henan China; 2 Henan Province Industrial Technology Research Institute of Resource and Materials, Zhengzhou University, Zhengzhou, 450000 Henan China; 3 Zhengzhou No.81 Middle School, Zhengzhou, 450000 Henan China

**Keywords:** Materials science, Nanoscale materials

## Abstract

Considering the problem of high oxygen content in industrial niobium powder, the oxygen reduction of high oxygen niobium powder with the addition of magnesium is studied. Based on the thermodynamic analysis of magnesium thermal reduction of niobium powder, the effects of reduction temperature, magnesium addition, reduction time, and reduction atmosphere on the oxygen content of pickling niobium powder are studied. The results show that with an increase in the magnesium addition, the oxygen content of pickling niobium powder gradually decreases to a certain value, and then remains unchanged. In a certain temperature range (953–1203 K), with an increase in the reduction temperature, the oxygen content of pickling niobium powder first decreases, and then increases; the best oxygen content is 356 ppm at 1133 K. With the extension in reduction time (2–6 h), the oxygen content of pickling niobium powder first decreases, and then remains unchanged. Finally, the oxygen content of pickled niobium powder is reduced to approximately 356 ppm at 400% magnesium addition at 1133 K for 4 h.

## Introduction

Niobium and niobium alloys are widely used in various industries, such as electronic, aerospace, iron, steel, automobile, and medical diagnostic equipment, owing to their good physical, mechanical, and electrical properties, such as good corrosion resistance and ductility, low density, high melting point, high specific strength, good electrical and thermal conductivities, as well as small thermal neutron capture cross section. Niobium powder is an important raw material for the preparation of niobium and niobium alloys. Currently, industrial niobium powder is obtained by crushing niobium strips, followed by hydrogenation. The particle size of niobium powder is relatively small, which is conducive to the compactness of powder metallurgy products. However, the niobium powder with small particle size has high affinity with oxygen and can adsorb oxygen, resulting in high oxygen content of the niobium powder^1–5^. Excessive oxygen content in niobium powder significantly affects its physical, mechanical, and electrical properties, restricting the application of niobium and niobium alloys^6–10^. Therefore, it is necessary to reduce the oxygen content in niobium powder.

Recently, the preparation of fine niobium powder with low oxygen content through magnesium reduction of Nb_2_O_5_ has attracted increased attention. Magnesium thermal reduction possess several advantages, such as simple process, low energy consumption, and easy separation of reaction products. However, there are only a few studies on magnesium reduction for reducing the oxygen content of niobium powder^11–15^. Even after oxygen reduction, the oxygen content of niobium powder was high, and the complete degree of oxygen binding with magnesium during magnesium reduction, as well as the degree of secondary oxidation on the surface of niobium powder during cooling, affected the oxygen content of niobium^15–22^.

In this study, the industrial niobium powder with 4100 ppm oxygen content and 9.8 µm Fisher particle size is studied. First, the thermodynamics and kinetics of magnesium thermal reduction of niobium powder are analysed to select the appropriate reduction temperature range. Based on this, the effects of magnesium addition, reduction time, reduction atmosphere, and reduction temperature, on the oxygen content of pickling niobium powder are studied. Finally, the influence of two-step reduction method on the oxygen content of pickling niobium powder is discussed.

## Results and discussion

### Magnesium addition

The amount of magnesium initially affects the degree of oxygen reduction on the surface of niobium powder^23^, and then affects the oxygen content in the pickling niobium powder. Moreover, magnesium addition affects the loose degree of reduced products, which further affects the pickling effect. Excessive magnesium causes niobium powder to be wrapped into a block by molten magnesium during cooling. The reduced products with serious sintering are not conducive to the acid pickling in later stages. When magnesium volatilises before the complete reduction of oxide on the niobium surface, leading to a high oxygen content in the pickling niobium powder, loose niobium powder is obtained. Therefore, we need to optimise the amount of magnesium.

**Figure 1:**
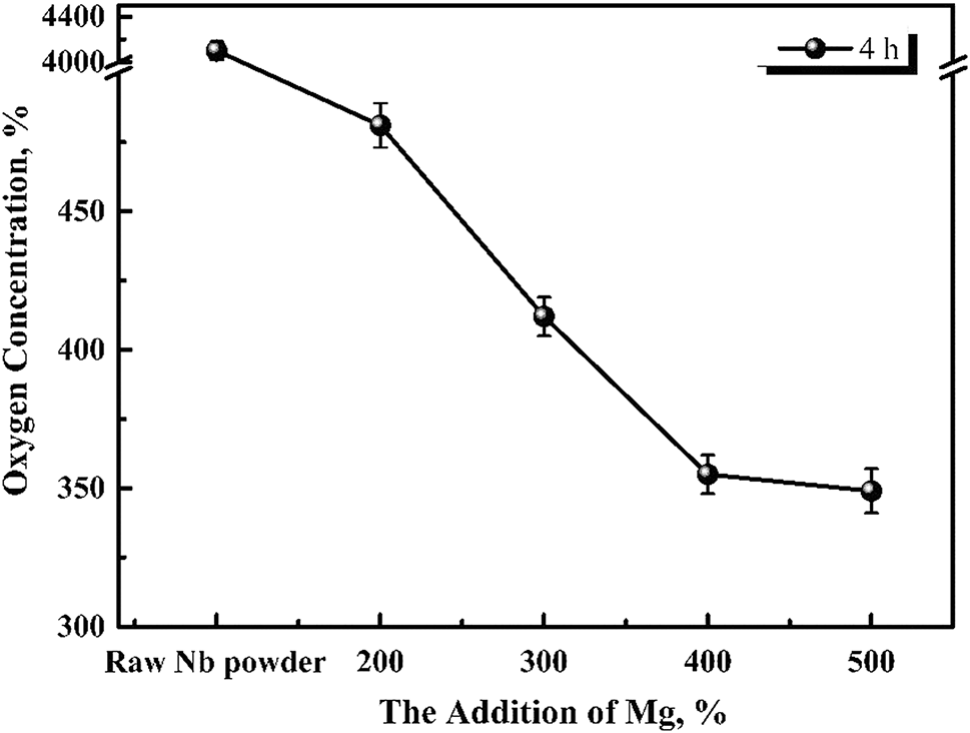
Effect of magnesium addition on the oxygen content of pickling niobium powder at 1133 K for 4 h.

**Figure 2:**
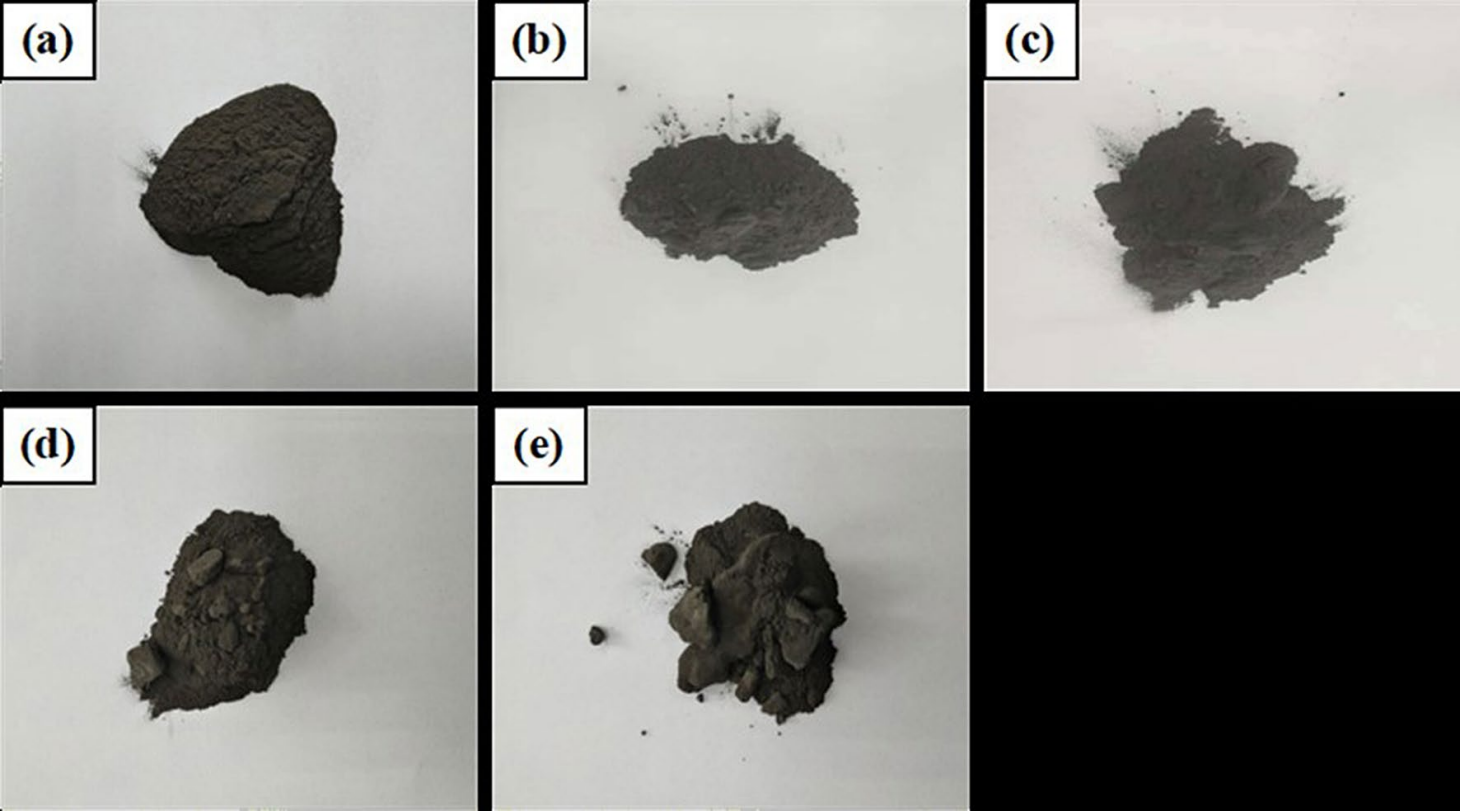
Morphology of reduced niobium powder with various amounts of magnesium additions. (**a**) Original niobium powder, (**b**) 200% theoretical amount, (**c**) 300% theoretical amount, (**d**) 400% theoretical amount, and (**e**) 500% theoretical amount.

The effects of 200, 300, 400, 500, and 600%, theoretical magnesium addition on the oxygen content of acid washed niobium powder were studied under argon reduction atmosphere in a graphite crucible at 1133 K for 4 h. The reduced product was vacuum dried at 333 K for 6 h, and the oxygen content was measured by ONH analyser. The change trend is shown in Fig. 1. The macro morphology of reduced products obtained with the original niobium powder and after magnesium addition is shown in Fig. 2.

As shown in Fig. 2b, when the original niobium powder (with high oxygen content) ,as shown in Fig. 2a, is reduced with 200% magnesium at 1133 K for 4 h, the reduced product is loose without any caking. A small amount (approximately 0.5%) of magnesium residue is found on the surface of niobium powder, and the oxygen content of niobium powder is 481 ppm after pickling. When magnesium addition is increased to 300%, some reduced products slightly agglomerate; however, the cohesion between them is negligible, which increases the residual magnesium chips, as shown in Fig. 2c. At this time, the oxygen content of acid washed niobium powder is approximately 412 ppm. With the increase in magnesium content to 400%, as shown in Fig. 2d, the reduced product forms and the hardness of the powder increases; however, the powder can be broken with a slight force, which does not cause difficulties in the subsequent pickling. At this time, the oxygen content of the pickling niobium powder is approximately 356 ppm. When the addition of magnesium is increased to 500%, as shown in Fig. 2e, many reduced products agglomerate. At this time, the oxygen content of the pickling niobium powder is 349 ppm; however, the hardness of the reduced products is high, which causes difficulties in the subsequent pickling. It can be concluded that, the oxygen content of pickling niobium powder is related to the loose degree and caking of reduced niobium powder. Under the reduction at 1133 K for 4 h, the oxygen content of pickled niobium powder decreases to the same level as with the increase in magnesium content from 200 to 500%. When the magnesium content is 400%, the loose degree of reduced niobium powder is the best, and the oxygen content of pickling niobium powder is the lowest, approximately 356 ppm. It can be concluded from the loose state of reduced products corresponding to different magnesium additions that, the agglomeration at 1133 K for 4 h is not due to the self-sintering of niobium powder (without magnesium addition), but due to the solidification during the cooling caused by excessive magnesium.

### Effect of reduction temperature and time on the oxygen content of pickling niobium powder

**Figure 3:**
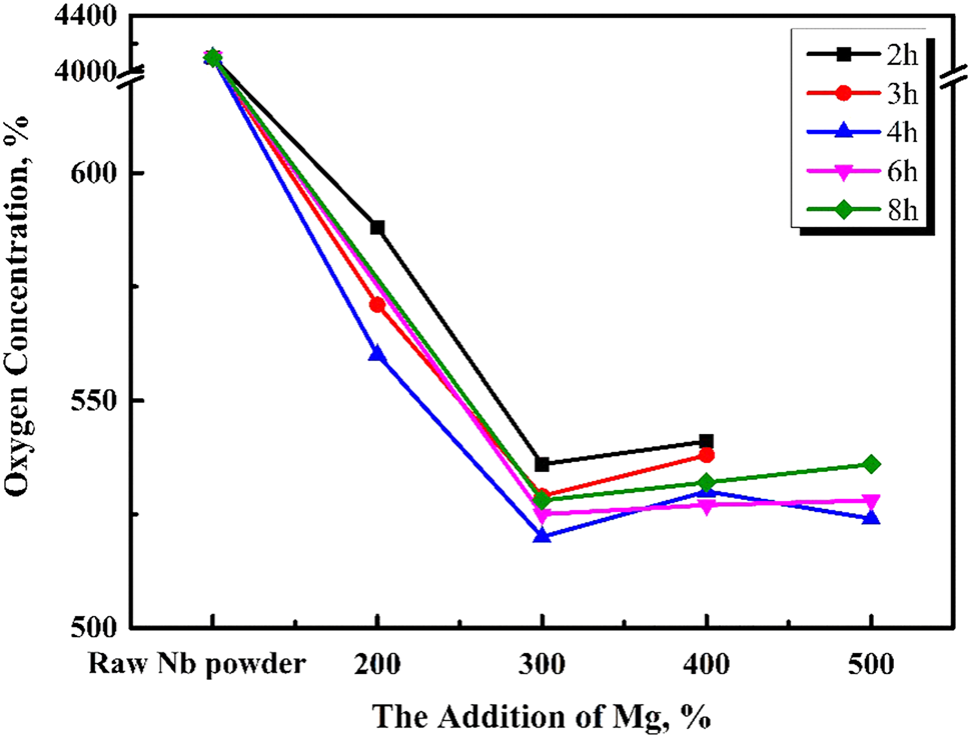
Effect of magnesium addition and reduction time on the oxygen content of pickling niobium powder at 1053 K.

The effects of reduction temperature and time on the oxygen content of acid washed niobium powder were studied under argon reduction atmosphere in a graphite crucible with optimum pickling and drying. The temperatures 953, 1053, 1093, and 1133 K, were selected. Then, the effects of various reduction times and the amounts of magnesium additions on the oxygen content of pickling niobium powder were studied at a specific reduction temperature. The results are shown in Figs. 3, 5, 6, and 7.

When the reduction temperature is 953 K and the reduction time is 8 h, with the increase in magnesium content, the oxygen content of pickling niobium powder initially decreases, and then remains constant, revealing that 200% magnesium addition is sufficient. According to the results, large particles of residual magnesium are observed on the surface of niobium powder after reduction, which indicates that magnesium thermal reduction is not thoroughly performed at this time. This is due to the low reduction temperature and slow reaction.

**Figure 4:**
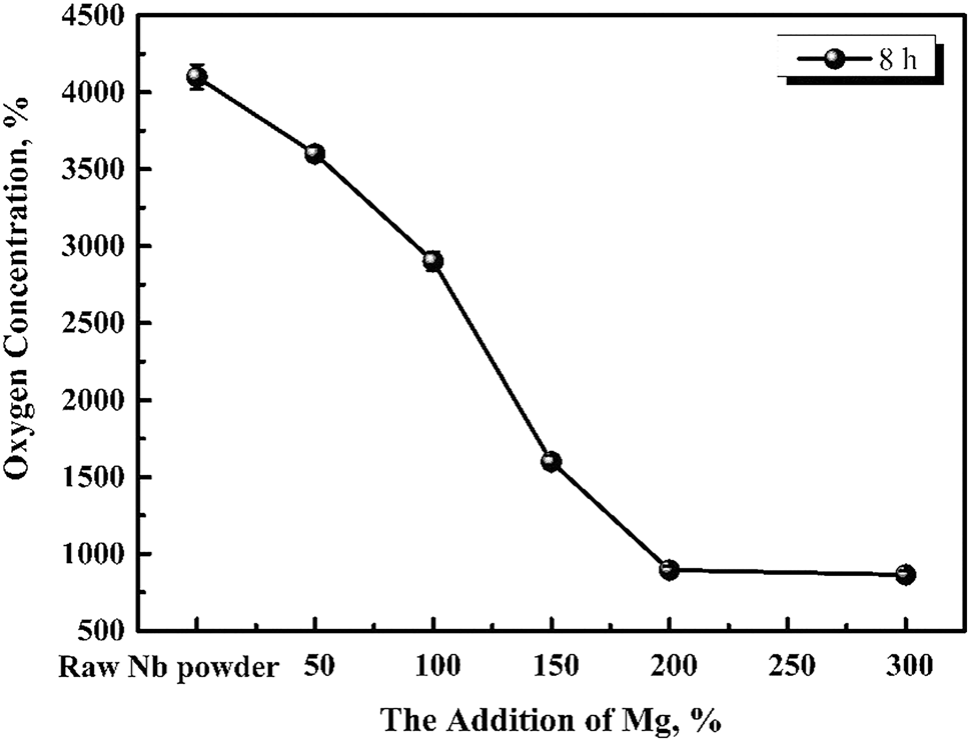
Oxygen content of pickling niobium powder at 953 K for 8 h with various amounts of magnesium additions.

**Figure 5:**
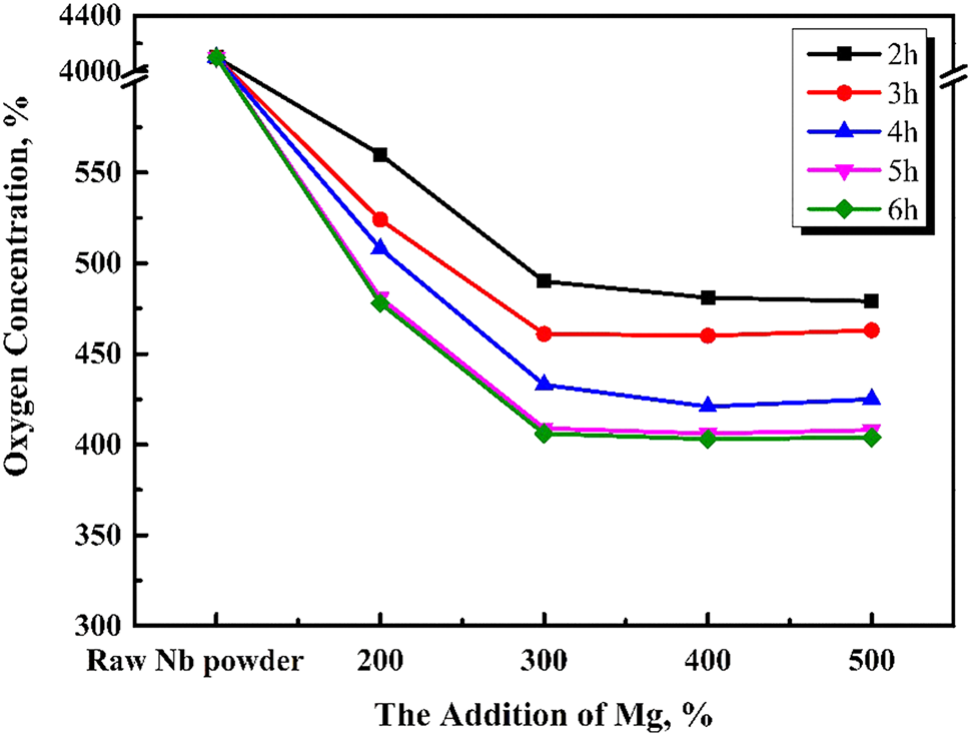
Effect of magnesium addition and reduction time on the oxygen content of pickling niobium powder at 1093 K.

According to Fig. 3, the oxygen content of pickling niobium powder is approximately 530 ppm when the magnesium content is 300% at 1053 K, and it is not affected by the reduction time. Compared to Fig. 4, the oxygen content of pickling niobium powder is obviously reduced. With the increase in reduction temperature, the reaction rate of magnesium reducing Nb_2_O_5_ accelerates, and the magnesium thermal reduction is relatively sufficient. When reduction time prolongs, the decrease in the oxygen content of acid washed niobium powder is insufficient, indicating that the reduction temperature of 1053 K and reaction speed are low. The results show that the oxygen content of pickling niobium powder with 200% magnesium addition is significantly higher than that with 300% magnesium addition. However, the oxygen content of pickling niobium powder with 400 and 500% magnesium addition negligibly changes compared to that with 300%. It shows that 300% excess magnesium is required to meet the magnesium consumption during the reduction at 1053 K, which is higher than that at 953 K. This is because the volatilisation loss of magnesium increases with increase in reduction temperature. Simultaneously, the addition of magnesium cannot completely assure the oxygen reduction at 1053 K (Fig. 5).

When the reduction time increases from 2 to 6 h, the oxygen content of pickling niobium powder first decreases, and then remains unchanged. The minimum oxygen content of the acid washed niobium powder is 406 ppm after 5 h reduction^15^. Owing to various experimental conditions, especially when the reduction temperature increases from 1053 to 1093 K, the oxygen content of niobium powder decreases from 530 to 406 ppm, which is also a reason for higher reduction temperature. The higher the reduction rate of magnesium to Nb_2_O_5_, the more effective the reduction is. When the magnesium content is more than 300%, the oxygen content of niobium powder is obviously lower than that of 200%. The oxygen content of niobium powder remains unchanged when the magnesium content increases to 400%. This shows that 300% magnesium addition is sufficient to consume magnesium at 1093 K reduction temperature. At 1093 K, the optimal parameters for magnesium reduction are 400% magnesium addition and 5 h pickling time. At this time, the oxygen content of niobium powder is 406 ppm.

**Figure 6:**
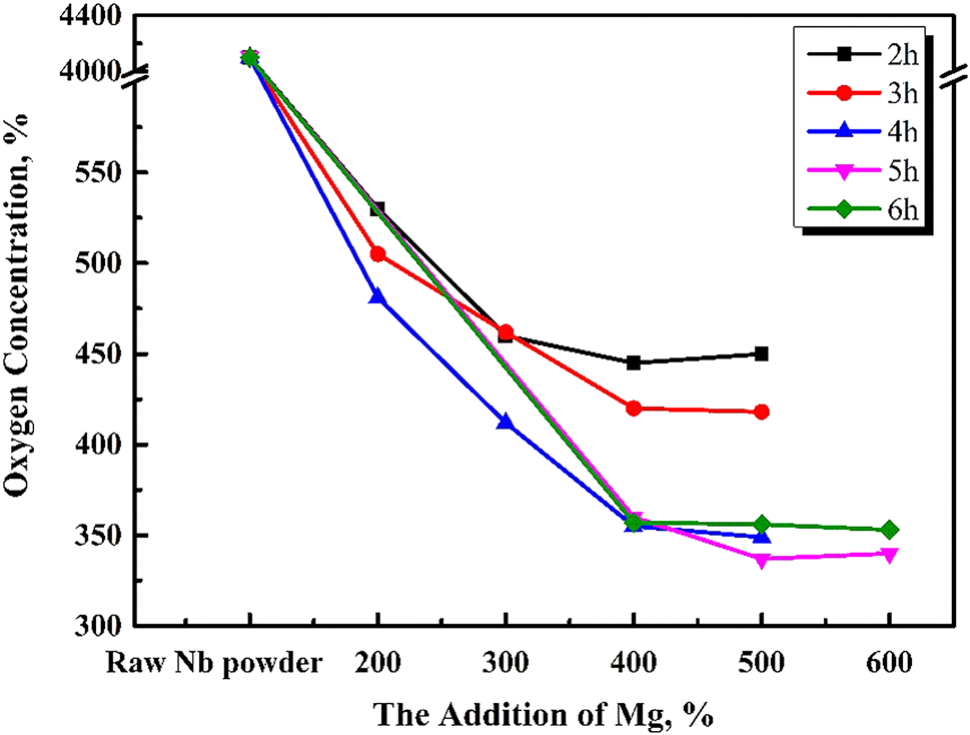
Effect of magnesium addition and reduction time on the oxygen content of pickling niobium powder at 1133 K.

As shown in Fig. 6, when the reduction temperature is 1133 K and the magnesium content is 400%, with the reduction time increasing from 2 to 6 h, the oxygen content of niobium powder first decreases and then remains unchanged. The happens because, the extension of reduction time makes the reduction of magnesium excessively effective. When the reduction temperature increases from 1093 to 1133 K, the oxygen content of pickling niobium powder decreases from 416 to 356 ppm, and the reduction time is 1 h shorter than 1093 K. The oxygen content of niobium impregnated powder is obviously lower than that of the niobium impregnated powder with the magnesium contents of 400, 200, and 300%. When the addition of magnesium increases to 500 and 600%, the oxygen content of niobium powder remains unchanged, and the reduced product can easily agglomerate. At 1133 K, the optimum magnesium reduction parameters are as follows: excessive magnesium addition of 400%, reduction time of 4 h, and oxygen content of 356 ppm.

**Figure 7:**
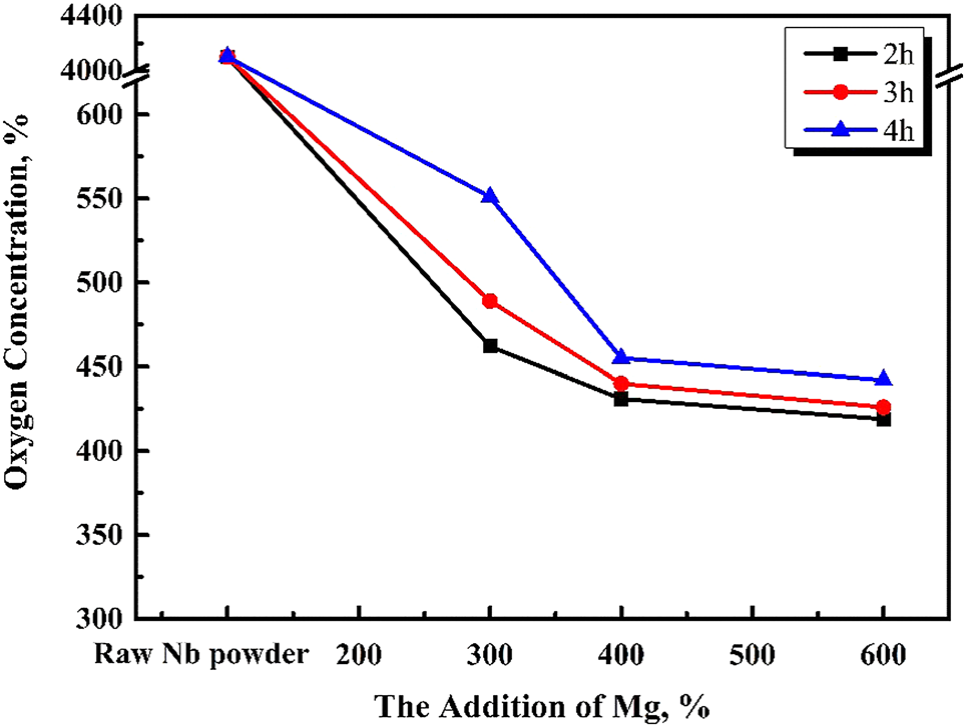
Effect of magnesium addition and reduction time at 1203 K on the oxygen content of pickling niobium powder.

According to the influence of the reduction temperature of 953, 1053, 1093, and 1133 K, on the oxygen content of pickling niobium powder, when the reduction temperature increases, the magnesium thermal reaction rate accelerates, making the reaction sufficient, and the pickling oxygen content decreases from 890 to 356 ppm. The effect of 1203 K reduction temperature on the oxygen content of pickling niobium powder is explored, and the results are shown in Fig. 7.

As shown in Fig. 7, the oxygen content of niobium powder at 1203 K is generally higher than that at 1133 K. Although the addition of magnesium increases from 300 to 600%, the oxygen content of niobium powder decreases. However, the reduced products with excess magnesium of 400, 500, and 600%, undergo caking and their hardness increases. A large amount of magnesium oxide and magnesium cause inconvenience in the subsequent pickling. The high reduction temperature of 1203 K is not conducive to the reduction of oxygen content in niobium powder. Therefore, the reduction process is optimised: reduction temperature is 1133 K, excess magnesium content is 400%, reduction time is 4 h, and the oxygen content of pickling niobium powder is 356 ppm.

**Figure 8:**
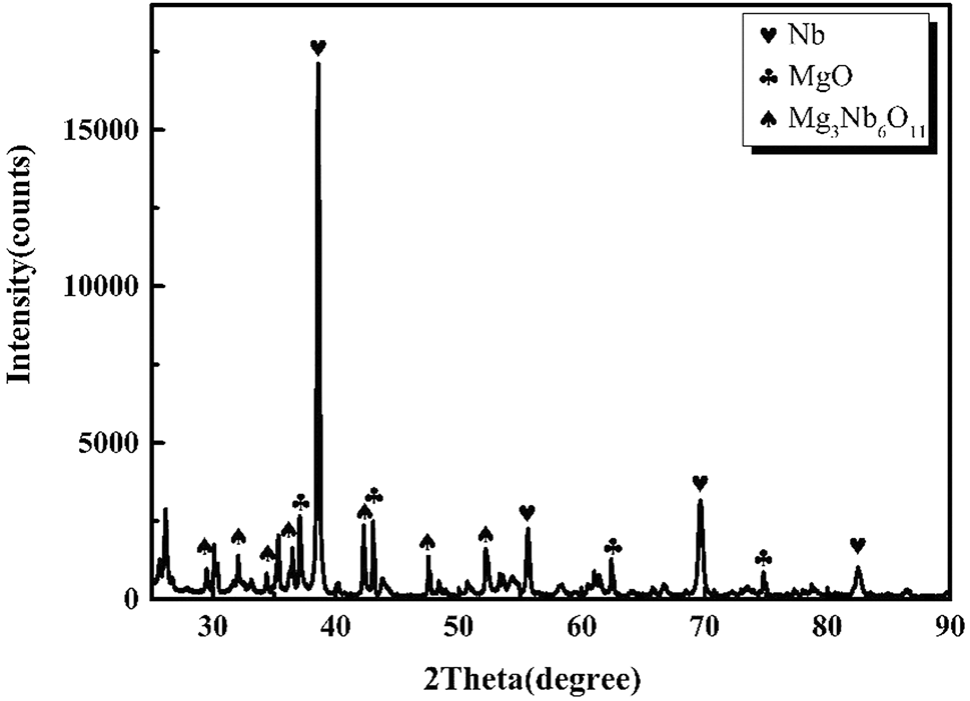
XRD pattern of the reduced products of magnesium at 1203 K for 2 h.

As shown in Figure 8, Mg_3_NbO_11_ is formed after the reduction at 1203 K for 2 h. The results show that the oxygen content of 1203 K reduced niobium powder after pickling is higher than 1133 K reduced niobium powder^15,24–26^. The oxygen content of niobium powder is significantly affected by the reduction temperature, reduction time, and magnesium content. The oxygen content of niobate powder decreases from 890 to 356 ppm. However, when the reduction temperature increases to 1203 K, the oxygen content of niobate powder increases owing to the existence of acid insoluble magnesium niobate in the reduced product.

The effect of reduction time on the oxygen content of pickling niobium powder is studied under efficient magnesium addition. At 1053 K for 2–8 h, the oxygen content of pickled niobium powder slightly changes, approximately 530 ppm. At 1093 K and 400% magnesium for 2–6 h, the oxygen content of pickled niobium powder decreases from 481 ppm in 2 h to 406 ppm in 5 h. At 1133 K for 6 h, the oxygen content of pickled niobium powder remains unchanged, approximately 403 ppm. The oxygen content of acid washed niobium powder decreases from 445 ppm in 2 h to 356 ppm in 4 h after 2–6 h reduction with 400% magnesium addition. The oxygen content of acid washed niobium powder changes from 356 to 357 ppm after 6 h reduction with increasing reduction time. Therefore, at 1093 and 1133 K, the oxygen content of pickling niobium powder first decreases, and then remains unchanged with increasing reduction time.

At the same reduction temperature and time, the oxygen content of pickling niobium powder first decreases, and then remains unchanged with the increase in magnesium content. The results show that the reduced products are relatively loose when the magnesium addition is 200 and 300%. When the magnesium addition is 400%, the reduced products obviously agglomerate, but they can be broken with a slight force. The reduced products are loose, and the oxygen content of pickling niobium powder is the lowest. When the magnesium addition is 500%, the reduced products agglomerate more, the hardness increases, and the oxygen content of pickling niobium powder is almost the same as that of 400% magnesium. Therefore, the optimal reduction parameters of magnesium reduction are optimised as follows: reduction temperature of 1133 K, reduction time of 4 h, and excess magnesium addition of 400%.

### Effect of reducing atmosphere and crucible material on the oxygen content and phase composition of pickling niobium powder

To explore the influence of reduction atmosphere and material of charging crucible on the oxygen content of acid washed niobium powder, the reduction at 1133 K with 400% magnesium addition for 4 h was performed. To study the influence of hydrogen, argon, and vacuum reduction atmosphere, as well as graphite and nickel crucible, on the oxygen content of acid washed niobium powder, the pressure of vacuum, argon and hydrogen are 0.1 Pa, 0.1 MPa, and 0.1 MPa, respectively. The oxygen content of acid washed niobium powder obtained by graphite and nickel crucibles under vacuum and argon reduction atmosphere are shown in Table 1.

**Table 1:** Oxygen content (PPM) of acid washed niobium powder reduced by different reduction atmospheres and crucibles.

Reducing atmosphere crucible material	Vacuum atmosphere	Argon atmosphere
Nickel crucible	764	371
Graphite crucible	802	365

As shown in Table 1, the oxygen content of niobium powder under vacuum reduction and pickling with two kinds of crucibles is higher than that under argon reduction. The main reasons are as follows: First, there is inevitable air infiltration in the hydrogenation and dehydrogenation furnaces under vacuum. During vacuum cooling, when the cooling temperature is 573 °C, the surface of niobium powder with high activity oxidises to niobium oxide. Hence, niobium powder oxidises even after oxygen reduction. Second, with the volatilisation of magnesium in vacuum and the decrease in temperature during cooling, the oxygen reduction ability of niobium powder gradually decreases. However, under high purity argon environment, the pressure in the furnace can be maintained at approximately 0.1 MPa, not allowing outside air in the furnace. Therefore, niobium powder with lower oxygen content can be easily obtained under argon atmosphere than under vacuum.

The niobium powder in graphite crucible during oxygen reduction is better because nickel crucible is covered during the reduction, and magnesium vapour always fills the whole nickel crucible. When graphite crucible is used without cover, the magnesium vapour rapidly volatilises, which is not conducive to the complete reduction of niobium powder. The addition of cover negligibly influences the final oxygen content. However, in the follow-up study, while using crucible, the cover can be considered.

**Figure 9:**
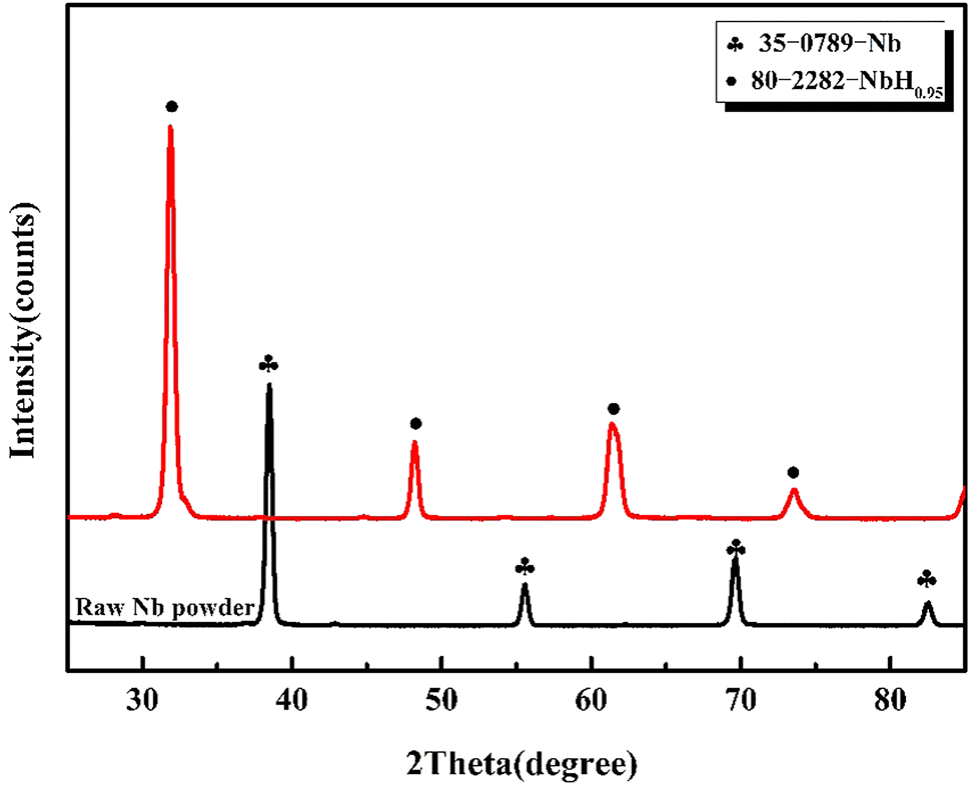
XRD diffraction pattern of niobium powder reduced in hydrogen medium.

Niobium powder exhibits good hydrogen absorption properties and can easily react with hydrogen to form brittle niobium hydride. Niobium has the fastest formation rate of NbH at approximately 633 K, and NbH begins to dehydrogenate at approximately 923 K. To investigate the hydrogen absorption of niobium powder during the reduction under hydrogen atmosphere, the reduction of niobium powder at 1133 K under hydrogen atmosphere is performed, and the reduced products are analysed by XRD diffraction. The results are shown in Fig. 9.

As shown in Fig. 9, under hydrogen atmosphere, the diffraction peak position of niobium powder after magnesium reduction is consistent with that of 80-2282-NbH0.95, indicating that niobium powder is completely transformed into NbH_0.95_ after the reduction under hydrogen atmosphere. Therefore, it is not suitable to reduce niobium powder in hydrogen state. At high temperature, nickel can easily form low melting point eutectic with magnesium, increasing the nickel content in pickling niobium powder. The carbon content of niobium powder can be increased by 70 ppm, when a carbon crucible is used. Therefore, it is necessary to re-select the crucible material in the future research.

### Effect of oxygen reduction by magnesium on the particle size, morphology, and solid solution oxygen of niobium powder

**Figure 10:**
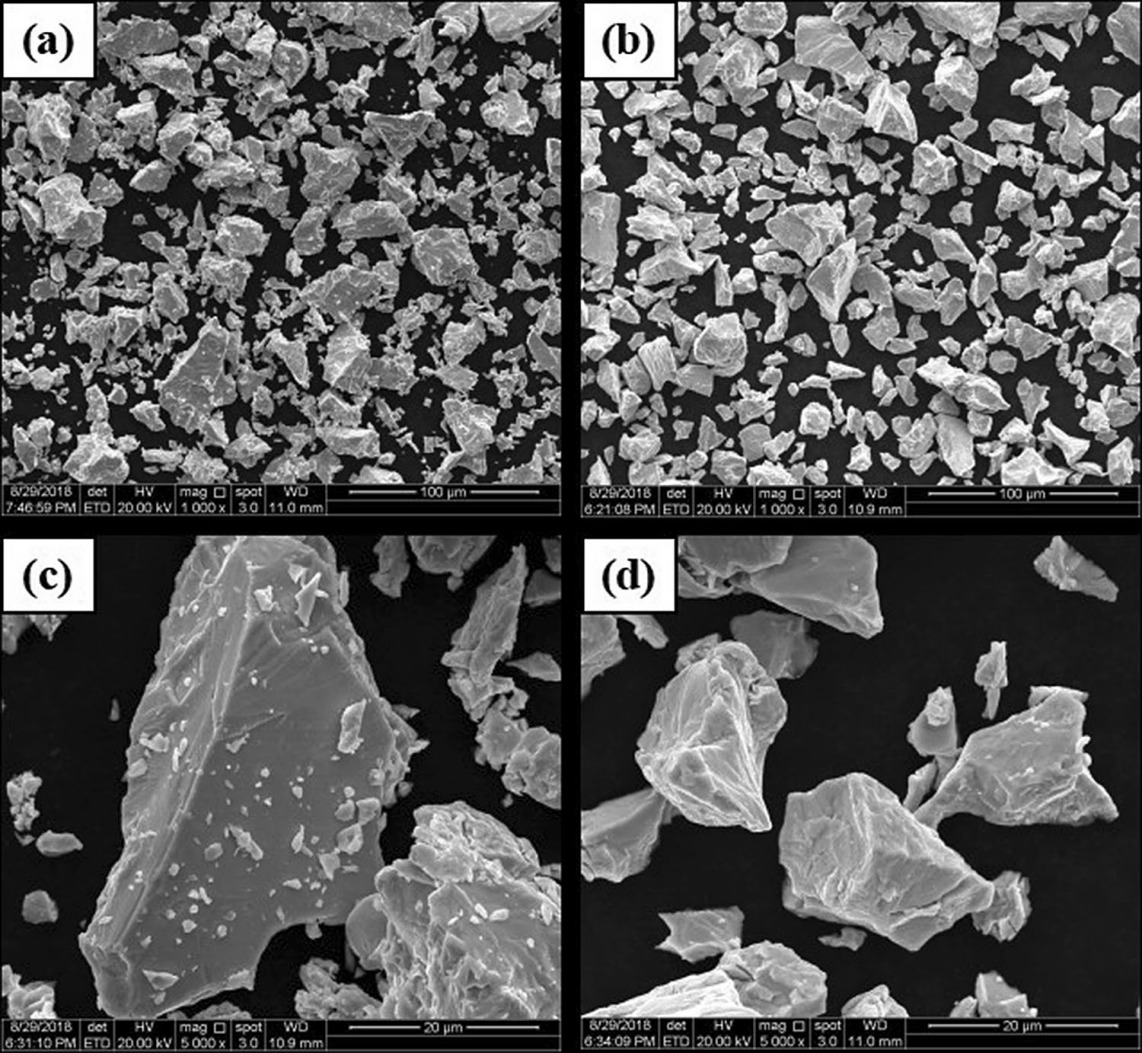
SEM images of niobium powder before and after oxygen reduction: (**a**) low rate before oxygen reduction, (**b**) low rate after oxygen reduction, (**c**) high rate before oxygen reduction, and (**d**) high rate after oxygen reduction.

**Figure 11:**
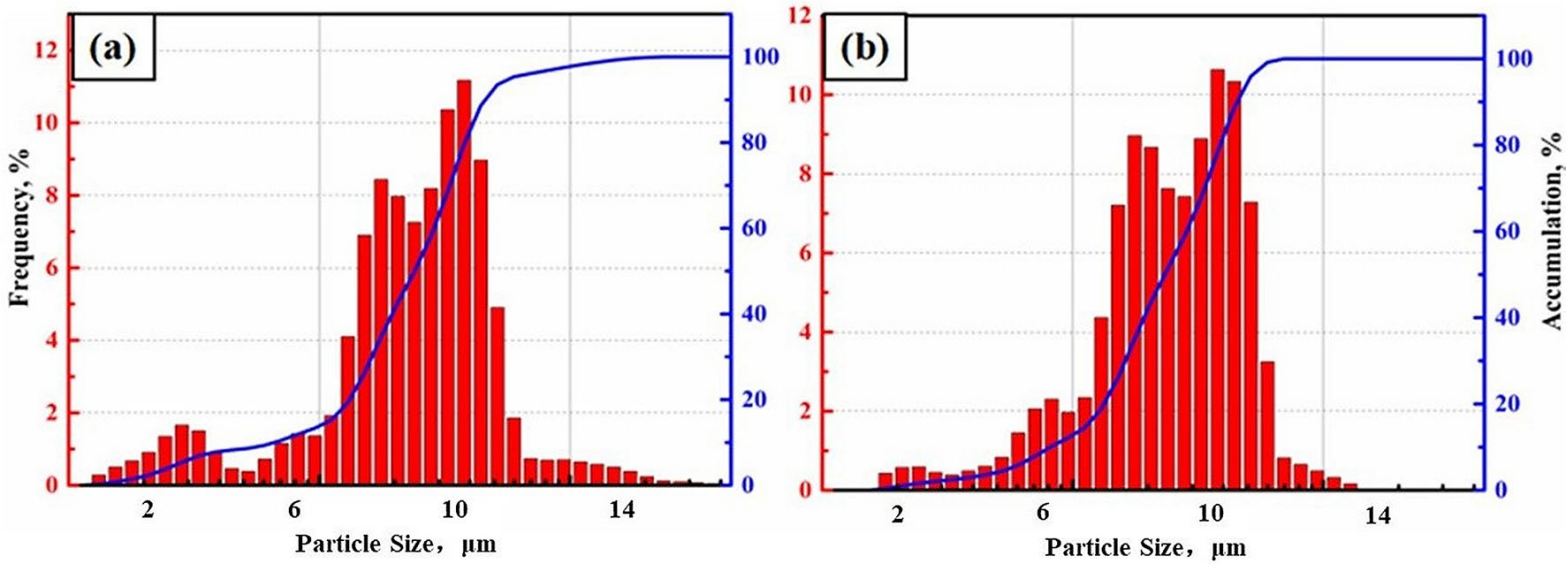
Particle size distribution of niobium powder before and after oxygen reduction: (**a**) before pickling and (**b**) after pickling.

To investigate the effect of reduction and pickling on the particle size and morphology of niobium powder, the particle size and morphology of the original niobium powder with high oxygen and the original niobium powder were studied at 1133 K temperature with 400% magnesium addition for 4 h. Figure 10 shows the SEM images of the original niobium powder and oxygen reduced niobium powder. Figure 11 is the particle size distribution of niobium powder before and after oxygen reduction.

As shown in Fig. 10a, the larger particle size of niobium powder before oxygen reduction is approximately 40 µm, and the smaller one is approximately 0.5 µm. As shown in Fig. 10c, the shape of niobium powder before oxygen reduction is irregular and sharp, and there are many fine particles of approximately 0.2–2 µm adsorbed on the surface of large particles. As shown in Fig. 10b, the minimum particle size of niobium powder after oxygen reduction is approximately 5 µm, and the maximum particle size is still approximately 40 µm, with no obvious change in morphology compared to that before deoxidation. Therefore, during reduction and pickling, the oxygen reduction of niobium powder does not affect its morphology. However, as shown in Fig. 10d, the niobium powder of fine particles significantly reduces after oxygen reduction, and the fine particles dissolve or wash away by the acid during pickling and water washing. The average particle sizes of original niobium powder and niobium powder after oxygen reduction are 9.8 and 10.2 µm respectively. The particle size distribution of niobium powder before and after oxygen reduction is shown in Fig. 11.

As shown in Fig. 11, oxygen reduction causes the loss of fine particles in niobium powder. According to calculation, the loss rate of niobium powder after the oxygen reduction is approximately 0.3%.

**Figure 12:**
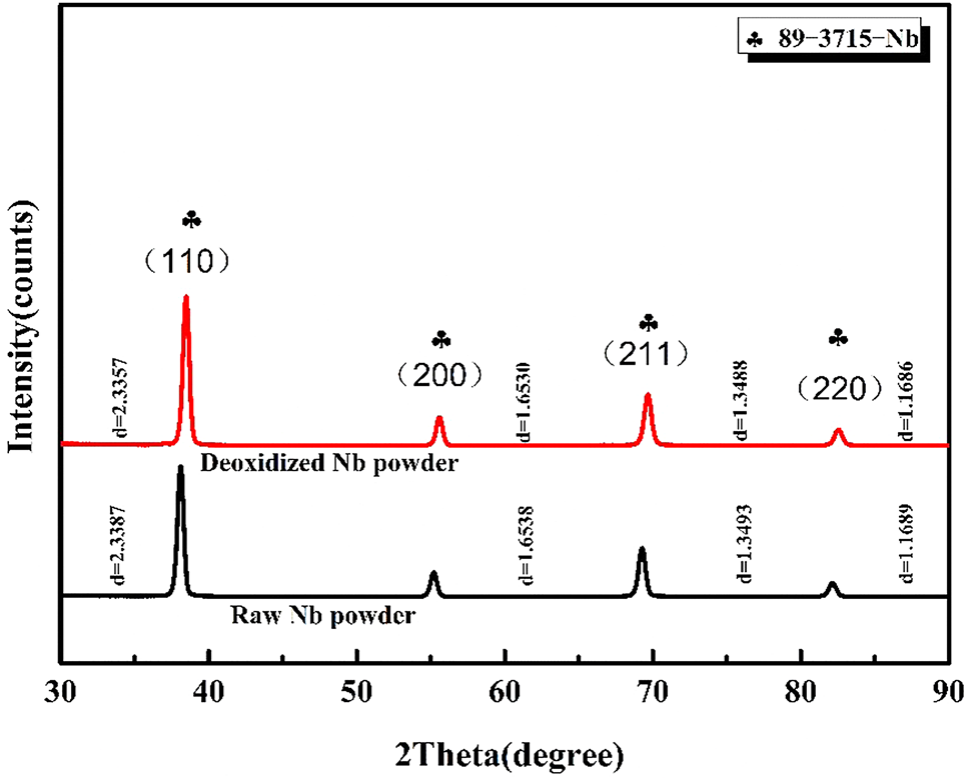
XRD diffraction pattern of niobium powder before and after deoxidisation.

To explore the effect of oxygen reduction on the dissolved oxygen in niobium powder, XRD analysis was performed on the acid washed niobium powder at 1133 K with 400% magnesium addition for 4 h. The diffraction pattern is shown in Fig. 12.

As shown in Fig. 12, no new phase is formed after the acid pickling of niobium powder reduced for 4 h at 1133 K with 400% magnesium addition, and only the single niobium phase remains. The peak shape of the diffraction peak is unchanged without obvious broadening. However, the crystal plane spacing of the niobium powder (110), (200), (211), and (220), before oxygen reduction tends to decrease compared to that after the oxygen reduction. For example, the (110) plane spacing D decreases from 2.3387 nm before oxygen reduction to 2.3357 nm after oxygen reduction, and the peak position angle shifts to the right by approximately 0.8°. Furthermore, oxygen reduction reduces the surface oxygen of niobium powder as well as solid dissolved oxygen. This is due to the lattice distortion of niobium caused by the oxygen dissolved in the niobium lattice. With the decrease in the oxygen content of niobium powder, the degree of the solid solution of oxygen in niobium decreases, decreasing the lattice distortion, that is, the decrease in the crystal plane spacing D. From Bragg’s equation,

1 $$ 2d\sin \theta = n\lambda $$

Here, N and λ are the reflection order and X-ray wavelength, respectively, D is the crystal plane spacing, and θ is the angle between the incident ray or reflected ray and the reflecting surface. When D decreases, θ increases. Therefore, the peak position of niobium powder after the oxygen reduction moves to the right, relative to the original niobium powder.

## Conclusion

In this study, the effects of magnesium addition, reduction temperature, reduction time, crucible material, and reduction atmosphere, on the oxygen content of niobium powder after pickling were studied. The main conclusions were as follows: The reduction temperature, magnesium addition, and reduction time, significantly influenced the oxygen content of acid washed niobium powder. When the reduction temperature increased from 953 to 1133 K, the oxygen content of niobium powder decreased from 890 to 356 ppm. When the reduction temperature increased to 1203 K, the oxygen content of niobium powder also increased. When the amount of magnesium was sufficient, the effect of reduction time on niobium powder varied according to the reduction temperature. When the reduction temperature was 1053 K and the reduction time was 2–8 h, the oxygen content of pickled niobium powder slightly varied, approximately 530 ppm. When the reduction time was 2–6 h at 1093 and 1133 K, the oxygen content of pickled niobium powder first decreased, and then remained unchanged from 481 to 406 ppm and from 445 to 357 ppm, respectively. At the same reduction temperature and time, the oxygen content of pickling niobium powder gradually decreased to a certain value with the increase in magnesium addition, and then remained unchanged. In addition, under argon atmosphere and graphite crucible, the oxygen content of niobium powder obtained by the oxygen reduction was better than that under vacuum atmosphere. The average particle size and morphology of the niobium powder after oxygen reduction were observed. Compared to that before oxygen reduction, there was almost no change in niobium powder, but a small amount of fine niobium powder was removed. After oxygen reduction, the peak angle of niobium powder shifted to the right by approximately 0.8 °C, resulting in the decrease in crystal plane spacing. Finally, when the reduction temperature was 1133 K, the excess amount of magnesium was 400%, reduction time was 4 h, and the oxygen content of pickling niobium powder reduced from 4100 to 356 ppm, which greatly reduced the oxygen content of industrial niobium powder.

## Experimental

### Materials

Hydrochloric acid, nitric acid, hydrogen peroxide, hydrofluoric acid, and analytically pure anhydrous ethanol, were purchased from Luoyang Haohua Chemical Reagent Co., Ltd (China). The purity of magnesium particles was 99.95% and the particle size was 1–10 mm, purchased from Aladdin Reagent Co., Ltd (USA). The niobium powder used in the experiment was provided by Ningxia Orient Tantalum Industry, with the oxygen content of 4100 ppm, particle size of 9.8 µm, and specific surface area of 0.2 m^2^/g. The specific chemical composition is shown in Table 2.

**Table 2:** Chemical composition of niobium powder.

Component	Nb	O	C	S	H	Fe	Si	Ti
Content/%	99.48	0.41	0.0203	0.005	0.002	0.013	0.057	0.011

### Sample preparation

The Nb_2_O_5_ reaction between magnesium powder and niobium surface $\left( {5Mg + Nb_{2} O_{5} = 2Nb + 5MgO} \right)$ produces niobium and MgO^22^. Theoretically, 1.5 g of magnesium is required for 1 g O. Since magnesium can easily volatilise owing to high saturated vapour pressure during sintering, and oxygen is inevitably introduced in the reduction atmosphere during the operation, the amount of magnesium should be excessive. In the early stage of experiment, the amount of magnesium added was 200, 300, 400, 500, and 600%, respectively. The meaning of excess is 2, 3, 4, 5, and 6 times, of the theoretical amount of magnesium, which is required for magnesium thermal reduction. The specific experimental method is as follows: 15 g niobium powder and corresponding amount of magnesium are evenly mixed and transferred into the crucible, and then reduced in the hydrogenation and dehydrogenation furnace. After natural cooling to room temperature, the furnace is opened for material collection, and acid washed. Further, drying and oxygen content analysis are performed.

**Figure 13:**
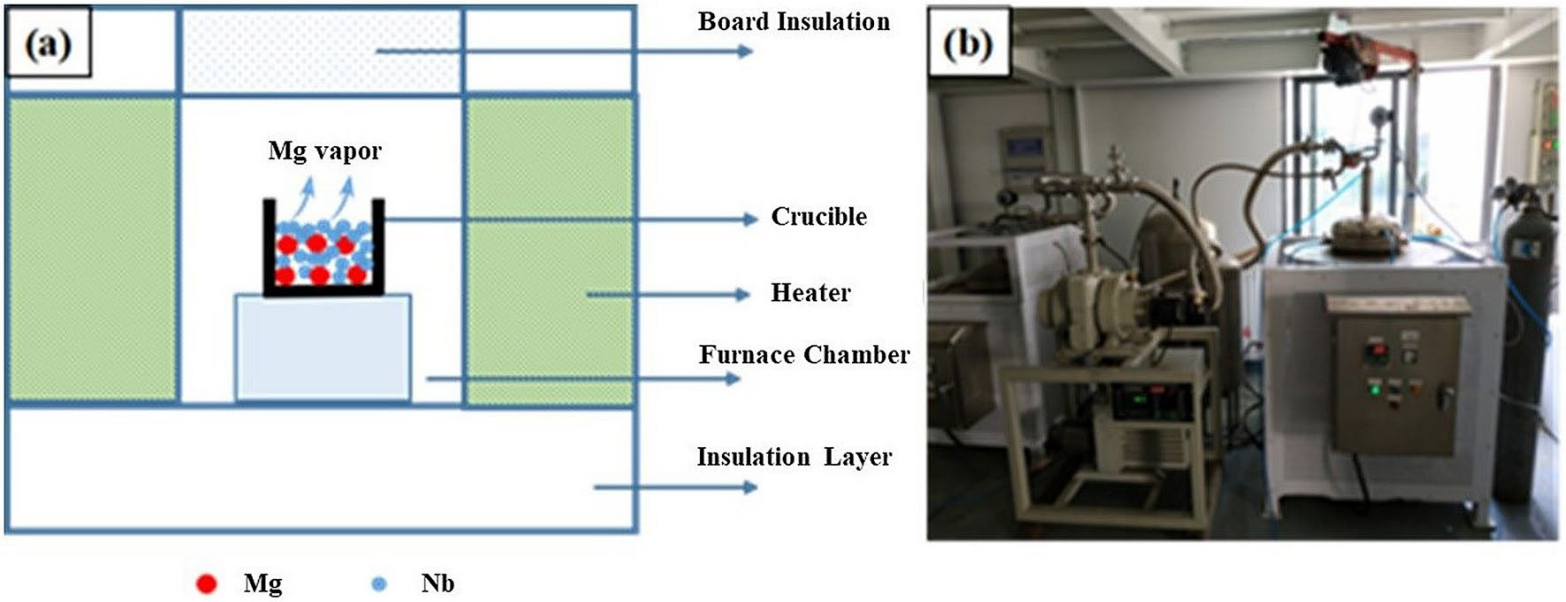
(**a**) Schematic of magnesium deoxidation charging and (**b**) equipment of magnesium deoxidation device.

The operation is as follows: the reaction chamber of the hydrogenation and dehydrogenation furnace is vacuumed below 0.1 Pa, purified argon id injected, then it is replaced three times, the oxygen content in the furnace is reduced, and the argon pressure of the protective gas in the furnace is set to 0.1 MPa. Then, the furnace is heated according to the heating procedure. During heating, owing to the gas expansion, the pressure in the furnace must be released at regular intervals to retain the gas pressure in the furnace at approximately 0.1 MPa. After heat preservation, it should be vacuumed for 1 h. Finally, according to the reduction temperature, appropriate amount of argon is injected under micro positive pressure to protect the cooling process. After natural cooling to room temperature, the furnace is opened for reclaiming, acid washing, drying, and oxygen content analysis. The equipment is self-designed. The schematic of magnesium deoxidation charging and equipment of magnesium deoxidation device are shown in Fig. 13.

Acid washing process: Using 37% HCl, 65% HNO_3_, 30% H_2_O_2_, and 40% HF, the pickling solution with 15% HCl + 1% H_2_O_2_ + 0.1% HF was prepared by volume ratio. The pickling was carried out in a magnetic stirrer with the solid–liquid ratio of 1:3, pickling temperature of 298 K, and pickling time of 100 min. Then, water washing was performed in ultrasonic wave until the pickling waste liquid was neutral. After the water on the surface of niobium powder was removed by suction filtration, it was washed twice with anhydrous alcohol for the quick evaporation of water. Then it was put in a glass dish and dried in vacuum oven. The oxygen content of the dried niobium powder was detected.

### Characterisation

The contents of oxygen, carbon, and other impurities, in niobium powder were determined by oxygen, nitrogen, and hydrogen content analyser (ONH-3000, Gangyannake Testing Technology Co., Ltd) as well as high frequency infrared carbon sulfur analyser (CS-3000, Gangyannake Testing Technology Co., Ltd). The metal impurities Fe, Ti, and non-metal impurities Si, in niobium powder were determined by inductively coupled plasma atomic analyser (ICP-AES, ThermoFisher Scientific). XRD (6100, Shimadzu) was used to analyse intermediate products during the reaction and detect any biological phase change. The experimental conditions are Cu-Kα rays, scanning speed of 2°/min, scanning angle of 30°–90°, working voltage of 40 kV, working current of 30 mA, and continuous scanning mode. Laser particle size analyser (Mastersizer 3000, Malvern) and average particle size analyser (WLLP-208a, Dandong Fisher Instrument Co., Ltd.) were used to determine the particle size of niobium powder. SEM and XPS were used to analyse the effect of oxygen on the morphology of niobium powder before and after oxygen reduction and the existing state of oxygen on the surface of original niobium powder. The morphology of niobium powder before and after oxygen reduction was characterised by field emission scanning electron microscope (Quant 250 FEG, USA Fei). The existence of oxygen on the surface of niobium powder with high oxygen content was analysed by X-ray photoelectron spectroscopy (AXIS Supra, Shimadzu).
