# Author Correction: Effect of magnesium reduction on the oxygen content of pickling niobium powder

**DOI:** 10.1038/s41598-021-98092-8

**Published:** 2021-09-08

**Authors:** Jingfeng Wang, Yue Zhang, Fang Liu, Qingkui Li

**Affiliations:** 1 Zhengzhou Railway Vocational and Technical College, Zhengzhou, 450000 Henan China; 2 Henan Province Industrial Technology Research Institute of Resource and Materials, Zhengzhou University, Zhengzhou, 450000 Henan China; 3 Zhengzhou No.81 Middle School, Zhengzhou, 450000 Henan China

Correction to: *Scientific Reports* 10.1038/s41598-021-94578-7, published online 22 July 2021

The original version of this Article contained an error in the spelling of the author Li Qingkui which was incorrectly given as Li Qinkui.

The original Article has been corrected.

